# Tripartite Data Analysis for Optimizing Telemedicine Operations: Evidence from Guizhou Province in China

**DOI:** 10.3390/ijerph17010375

**Published:** 2020-01-06

**Authors:** Jinna Yu, Tingting Zhang, Zhen Liu, Assem Abu Hatab, Jing Lan

**Affiliations:** 1 Business School, Guizhou Minzu University, Guiyang 550025, China; yujinna2004@163.com; 2 School of Economics and Management, University of Science and Technology Beijing, Beijing 100083, China; tzhang@ustb.edu.cn; 3 School of Business, Nanjing Normal University, Nanjing 210023, China; zhenliu_cn@yahoo.com; 4 Department of Economics, Swedish University of Agricultural Sciences, P.O. Box 7013, SE-750 07 Uppsala, Sweden; assem.abouhatab@slu.se; 5 Department of Economics & Rural Development, Arish University, Al-Arish 45511, North Sinai, Egypt; 6 College of Public Administration, Nanjing Agricultural University, Nanjing 210095, China

**Keywords:** telemedicine, tripartite data analysis, optimizing telemedicine operations

## Abstract

Telemedicine is an innovative approach that helps alleviate the health disparity in developing countries and improve health service accessibility, affordability, and quality. Few studies have focused on the social and organizational issues involved in telemedicine, despite in-depth studies of and significant improvements in these technologies. This paper used evolutionary game theory to analyze behavioral strategies and their dynamic evolution in the implementation and operation of telemedicine. Further, numerical simulation was carried out to develop management strategies for promoting telemedicine as a new way of delivering health services. The results showed that: (1) When the benefits are greater than the costs, the higher medical institutions (HMIs), primary medical institutions (PMIs), and patients positively promote telemedicine with benign interactions; (2) when the costs are greater than the benefits, the stability strategy of HMIs, PMIs, and patients is, respectively, ‘no efforts’, ‘no efforts’, and ‘non-acceptance’; and (3) promotion of telemedicine is influenced by the initial probability of the ‘HMI efforts’, ‘PMI efforts’, and ‘patients’ acceptance’ strategy chosen by the three stakeholders, telemedicine costs, and the reimbursement ratio of such costs. Based on theoretical analysis, in order to verify the theoretical model, this paper introduces the case study of a telemedicine system integrated with health resources at provincial, municipal, county, and township level in Guizhou. The findings of the case study were consistent with the theoretical analysis. Therefore, the central Chinese government and local governments should pay attention to the running cost of MIs and provide financial support when the costs are greater than the benefits. At the same time, the government should raise awareness of telemedicine and increase participation by all three stakeholders. Lastly, in order to promote telemedicine effectively, it is recommended that telemedicine services are incorporated within the scope of medical insurance and the optimal reimbursement ratio is used.

## 1. Introduction

Telemedicine is widely used in global health systems, especially in developing countries, as a new way to deliver healthcare services. China is the largest developing country in the world, 70% of its population live in rural areas, and there are serious disparities in medical resources between rural areas and cities [1]. Therefore, telemedicine is regarded as an important policy tool to narrow health disparities. In recent years, telemedicine in China has developed rapidly as telecommunication networks have expanded [1]. After several years of development, however, the adoption of telemedicine into mainstream health services has been slower than expected [2]. Telemedicine adapts conventional medical practice to enable patients to access medical services via telecommunication. Telemedicine has therefore established a new kind of relationship between smaller hospitals and larger ones, as well as between patients and hospitals in general. Patients and primary hospitals benefit from the resources of larger hospitals via digital tele-consultation, digital tele-diagnosis, and digital tele-monitoring. This is particularly beneficial for patients living in rural areas where the healthcare system is less developed than in cities. However, the interests of each side in telemedicine need to be balanced in order to promote the sustainable development of telemedicine in future. It is therefore appropriate to analyze the behavior of stakeholders in the telemedicine system to improve the promotion of telemedicine. In this study, evolutionary game theory was applied to analyze the behaviors and evolutionary framework of HMIs, PMIs, and patients, as well as theoretically explore whether the tripartite game will achieve an evolutionary stability strategy or only realize local stability. A numerical simulation was used to provide suggestions for the promotion and development of telemedicine and lay the foundation for a ‘hierarchical medical system’ in China.

## 2. Literature Reviews

Telemedicine has been researched for more than a century, but there is a lack of clarity and an absence of agreement about the definitions of key concepts in relation to the terminology in this field [3]. To date, although there is no single, commonly accepted definition of telemedicine, the use of technology to deliver healthcare services and information at a distance to improve access and quality and reduce costs is a common theme throughout professional descriptions of these services [4].

In developing countries in particular, telemedicine can help address the shortage of health workers and medical specialists by providing timely access to specialist and other forms of healthcare [5]. Recognizing the extreme shortage in health professionals in developing countries [6], a major challenge for promoting telemedicine is whether clinicians are willing to use telemedicine technology. Chau and Hu [7] examined physicians’ acceptance of telemedicine technology and suggest that the technology acceptance model (TAM) may be more appropriate than the theory of planned behavior (TPB) model for examining technology acceptance by individual professionals. As an extension of TAM, the UTAUT (Unified Theory of Acceptance and Use of Technology) model, which overcomes the limitations of the early TAM models, was adopted to explore the adoption and usage of telemedicine among clinicians in Nigeria [8]. Furthermore, existing literature also focuses on acceptance and the influencing factors of other entities, e.g., hospitals [9], technology suppliers, and service providers [10], whose research shows that the most frequently reported patient barriers to telehealth acceptance are technical issues. The technology supplier and service providers were thus able to design and deliver higher quality telemedicine services, adopting a user-centric approach. However, they are not the only ones involved. Patients’ willingness to use telemedicine is also crucial for expanding telemedicine’s usage due to increased demand. Previous telemedicine research has concentrated on technology development and technology acceptance from the standpoint of either an organization or a healthcare professional, hence, offering limited insights for patients’ acceptance of this technology. In this context, Liu [11] uses a quantitative survey research to address pervasive tele-monitoring acceptance among patients Gorst et al. [12] assess the levels of uptake of home telehealth by patients diagnosed with heart failure and chronic obstructive pulmonary diseases and the factors determining patients’ willingness to continue using telehealth. Moreover, Domingo et al. [13] explore the acceptance of telemedicine and its impact on patient behavior.

Researchers have tended to focus on the evaluation of telemedicine services, the mainstream view being that telemedicine has advantages over traditional medicine, such as saving patients substantial time and money with less travel [14], delivering greater continuity of care and patient satisfaction [14], and yielding better clinical results [15,16]. Telemedicine may also reduce pollution and the greenhouse gases associated with travel to hospitals [17], as well as reduce apprehension regarding sexual and reproductive health consultations [18]. That is not to say however that all researchers have found the same positive results. Some studies have even concluded that telemedicine does not seem to be a cost-effective addition to standard support and treatment [19].

In the existing studies evaluating telemedicine, researchers have neglected the role of healthcare providers and only focused on the effectiveness and satisfaction of patients using telemedicine [20]. This preference should be addressed in future research.

## 3. Methods

By and large, modelling human decision-making processes or actual human behavior is harder than that of the transparent physical systems dealt by traditional science and engineering, because the governing mathematical models are usually unknown. What we can guess concerning these processes is not expressed as a set of transparent, deterministic, and explicit equations but black box-like models or, in some cases, stochastic models. At any rate, in order to solve those problems in the real world, we must build a holistic model that covers not only environment as physical systems, but also human beings and society as complex systems. One effective tool to do this is evolutionary game theory [21].

### 3.1. Game Theory in Health

The concept of game theory has been used among the access to the healthcare services. Game theory has the potential to provide models of the consultation that can be used to generate empirically testable predictions about the factors that promote quality of care. In the research of Tarrant et al. [22], they indicated that game theory can been applied to the medical consultation and used to generate predictions about how the context of a doctor–patient interaction influences cooperation and quality of care, and further empirical work is needed to uncover the underlying game structures that occur most commonly in medical consultations. Blake and Carroll [23] analyze how game theory can provide a framework for understanding the strategic decision-making that occurs in everyday scenarios in medical training and practice, and ultimately serves as a tool for improving the work environment and patient care, through analyses, they found that trainees and physicians can work to better recognize where competing priorities exist, understand the motivations and interactions of the various players, and learn to adjust their approaches in order to ‘change the game’ when their preferred outcome is not the most likely one. Dasgupta et al. [24] describe a mixed reality-based testbed and a framework for studying social interactions using a game theory approach and present a case study for exposure therapy treatment of individuals with Social Anxiety Disorder using the proposed testbed. Based on multi-player cooperation game theory, Kumar et al. [25] propose a novel E-healthcare model coupled with cloud computing platform to provide health related services in IoV (Internet of Vehicles) environment on-the-fly. Through these studies, game theory has the potential to provide a new conceptual and theoretical basis for future empirical work on the interaction between doctors and their patients.

### 3.2. Evolutionary Game Theory and ESS

Evolutionary game theory (e.g., Weibull 1995 [26]) has evolved by merging game theory with the basic concept of Darwinism so as to compensate for the idea of time evolution, which is partially lacking in the original game theory that primarily deals with equilibrium. In our study, the evolution refers to the strategies adopted by the agents and, in most agent-based models, the mechanism responsible for this evolution is a process usually defined ‘strategy revision phase’. The latter allows agents to change their strategy according to a particular rule, where usually ‘rationality’ constitutes the main ingredient [27]. Maynard Smith [28] formulated a central concept of evolutionary game theory called the evolutionarily stable strategy (ESS). To briefly illustrate this, suppose that a population consists of individuals adopting strategies I or J with frequencies p and q, where p+q=1. What is the fitness of an individual adopting strategy?

$$\mathrm{Fitness}\;\mathrm{of}\;I=p\cdot E_{I}\left(I\right)+q\cdot E_{J}\left(I\right)$$

$$\mathrm{Fitness}\;\mathrm{of}\;J=p\cdot E_{I}\left(J\right)+q\cdot E_{J}\left(J\right)$$

If a particular strategy, say I, is to be an ESS, it must have the following property. A population of individuals playing I must be ‘protected’ against invasion by any mutant strategy, say J. That is, when I is common, it must be fitter than any mutant. That is, I is an ESS if for all J≠I,

$$either\;E_{I}\left(I\right)>E_{I}\left(J\right),$$

$$or\;E_{I}\left(I\right)=E_{I}\left(J\right),$$

$$and\;E_{J}\left(I\right)>E_{J}\left(J\right).$$

If these conditions are satisfied, then a population of individuals playing I is stable; no mutant can establish itself in such a population. This follows from the fact that when q is small, the fitness of I is greater than the fitness of J.

In research of Zeeman [29], an ESS is an attractor of the replicator dynamics, and the population converges to the ESS for every strategy sufficiently close to it. If I is an internal ESS, then global convergence to I is assured.

### 3.3. Data Collection

In order to comprehend the implementation and operation of telemedicine in poor Guizhou Province, our team conducted a social survey in Qiandongnan Miao and Dong Autonomous Prefecture in Guizhou between 7 and 22 February 2018. This field work focused primarily on the opinion, attitude, and behavior of rural residents regarding telemedicine. Interviews were conducted with the head of the township and county hospitals. In the survey, residents of 10 administrative villages (Leye Village, Longma Village, Anping Village, Longtian Community, Zongyuan Village, Luxi Village, Daidian Village, Juntun Village, Hujia Village, and Dusu Village) were samples under the jurisdiction of the town of Longtian. Furthermore, 10 households were randomly sampled from each village, resulting in a total sample of 100 observations. For the interviews, we only get information on the implement and operation of telemedicine, and the key issues during the telemedicine. With an estimated Cronbach’s Alpha of 0.547, the information we collected through the questionnaire as well as the interviews can be considered as moderately reliable. In addition, data were also collected from policy documents, reports, and news with regard to telemedicine in Guizhou Province. Our filed survey data as well as the secondary information together suggest the validity and rationality of the theoretical model and the numerical simulation of this paper.

## 4. Tripartite Evolutionary Game Model of Stakeholders in Telemedicine

### 4.1. The Hypothesis of the Tripartite Evolutionary Game Model

In China, MIs are required to sign telemedicine cooperation agreements to provide telemedicine services. The content of the agreements should include the purpose of the cooperation, its conditions, contents, the process of providing telemedicine services, the rights and obligations of both parties, the risks and liabilities involved in sharing responsibility for medical damages, etc. Telemedicine service fees are settled regularly by means of special financial accounts. It is strictly prohibited to obtain funds for telemedicine services in any other way. The daily operational costs of telemedicine in MIs includes expert diagnosis and treatment fees, training fees, site fees, equipment fees, network fees, operation and maintenance fees, management fees, full-time personnel fees, technical maintenance fees, and system maintenance fees. Here, HMIs mainly refer to hospitals at or above county level, while primary MIs mainly refer to township hospitals.

In China, telemedicine is a complex system that involves many services; thus, it is impossible to analyze each item in detail given the limited space of this paper. To simplify the analysis without compromising its accuracy, this paper only considers the treatment provided through telemedicine, e.g., tele-consultation, tele-diagnosis, and tele-monitoring. The following hypotheses were tested in this study:

(1) The promotion of telemedicine is influenced by the behavior of three groups: Inviters of telemedicine (mainly the primary MIs), the invitees of telemedicine (HMIs), and patients. All the players with bounded rationality can adjust their own strategies by imitating and learning proven behaviors to guarantee maximum revenues.

(2) When providing telemedicine, HMIs take two courses of action: One strategy is to prioritize the provision of telemedicine (showing effort), while the other is to provide telemedicine without prioritizing it (not showing effort). Thus, the strategy space of HMIs is $S_{1}\left\{\mathrm{efforts},\;\mathrm{no}\;\mathrm{efforts}\right\}$. Likewise, the strategy space of primary MIs and patients are $S_{2}\left\{\mathrm{efforts},\;\mathrm{no}\;\mathrm{efforts}\right\}$ and $S_{3}\left\{\mathrm{acceptance},\;\mathrm{non}-\mathrm{acceptance}\right\}$ respectively. Meanwhile, it is assumed that the efforts of HMIs are $e_{1}$ and those of primary MIs are $e_{2}$. It is also assumed that the efforts of HMIs and PMIs are independent of each other, and that the range of the efforts values is $\left[{\underline{e}}_{i},{\bar{e}}_{i}\right]$, where i=1,2.

(3) The tripartite game is a cooperative game without rent-seeking behaviors, i.e., the three groups do not collude or collaborate with one other. It is an asymmetric game. The assumptions are that HMIs may with x probability adopt the ‘efforts’ strategy, and with (1−x) probability adopt the ‘no efforts’ strategy; primary MIs may with y probability adopt the ‘efforts’ strategy, and with (1−y) probability adopt the ‘no efforts’ strategy; patients may with z probability adopt the ‘acceptance’ strategy, and with (1−z) probability adopt the ‘non-acceptance’ strategy, in which 0<x<1, 0<y<1, and 0<z<1, respectively.

(4) The payment of HMIs is $E_{1}\left({\bar{e}}_{1}\right)$ when they make efforts to provide telemedicine, while the corresponding operating costs are $C_{1}\left({\bar{e}}_{1}\right)$. In contrast, the payment and operating costs are $E_{1}\left({\underline{e}}_{1}\right)$ and $C_{1}\left({\underline{e}}_{1}\right)$, respectively, when HMIs do not make efforts to provide telemedicine. Since government funds support HMIs in the provision of telemedicine, they are based on patient satisfaction assessments and distributed through the post payment system; moreover, the level of financial support is a function of effort. In the aforementioned situation, the levels of government support are $R_{1}\left({\bar{e}}_{1}\right)$ and $R_{1}\left({\underline{e}}_{1}\right)$ respectively.

(5) In the process of the cooperative use of telemedicine between primary MIs and HMIs, there are two situations: (1) Where primary MIs make an effort to cooperate with HMIs, where the benefits and operation costs are $E_{2}\left({\bar{e}}_{2}\right)$ and $C_{2}\left({\bar{e}}_{2}\right)$, respectively, and the financial support provided by government is $R_{2}\left({\bar{e}}_{2}\right)$; and (2) where primary MIs do not try to cooperate with HMIs; in this situation, the benefits and operational costs are $E_{2}\left({\underline{e}}_{2}\right)$ and $C_{2}\left({\underline{e}}_{2}\right)$, respectively, and the financial support provided by the government is $R_{2}\left({\underline{e}}_{2}\right)$. In either situation, if primary MIs seek help from HMIs, they need to pay a fee to the HMIs according to their cooperative agreement. According to the telemedicine regulations in many provinces, the fee is a proportion of the total telemedicine expenses B paid by the patients. In this paper, it was assumed that the proportion is ε(0<ε<1) and is decided by both sides through consultation according to the government’s guidance price.

(6) When patients accept telemedicine services, their utility depends on the effort level *e_i_* of the HMIs and PMIs, indexed as $E_{3}\left(e_{1},e_{2}\right)$. Considering the range of HMIs and primary MIs efforts, the patients’ utility set is defined as {$E\left(\bar{e_{1}},\bar{e_{2}}\right)$, $E\left(\bar{e_{1}},\underline{e_{2}}\right)$, $E\left(\underline{e_{1}},\bar{e_{2}}\right)$, $E\left(\underline{e_{1}},\underline{e_{2}}\right)$}. To simplify the calculation, it was assumed that $E_{3}\left({\bar{e}}_{1},{\underline{e}}_{2}\right)=E_{3}\left({\underline{e}}_{1},{\bar{e}}_{2}\right)$. Furthermore, during the process of telemedicine, the utility of patients as telemedicine terminal mostly relies on the efforts of primary MIs that are agent of both HMIs and patients. Regardless of the level of HMIs efforts, patients would not get better telemedicine services if the primary MIs hold less or none efforts. Besides, if the HMIs negatively provide telemedicine service, even though the primary MIs put more efforts in the process of telemedicine, the utility of patients would still be low. Accordingly, the following simplified assumption is drawn: $E\left(\bar{e_{2}}\right)=E\left(\bar{e_{1}},\bar{e_{2}}\right)$, which implies a better utility of patients through telemedicine, and others of patients’ utility set are all equal to $E\left(\underline{e_{2}}\right)$, which represents a worse utility of patients through telemedicine. The telemedicine fee the patients pay is B once they accept the telemedicine service, regardless of the utility level. Assuming that the reimbursement ratio of New Rural Cooperative Medical Insurance (which has been incorporated into the basic medical insurance for urban and rural residents) is b(0<b<1), if telemedicine services are under the social medical insurance scheme, the real telemedicine fees that patients need to pay are (1−b)B. If telemedicine services are not incorporated into the social medical insurance scheme, then b=0. In addition, complaint costs G must be paid if patients are not satisfied with the telemedicine they accepted. When patients do not accept telemedicine, then the utility level and costs are both zero.

### 4.2. Payoff Matrix of the tripartite Evolutionary Game in Telemedicine

Based on the above assumptions, a tripartite evolutionary game model including HMIs, primary Mis, and patients under bounded rationality was constructed. The payoff matrix of the three groups is shown in Table 1.

## 5. Analysis of the Tripartite Evolutionary Game Model in Telemedicine

### 5.1. Replicator Dynamics Equation of the Tripartite Evolutionary Game

Under the aforementioned assumption, the marginal expected revenue when HMIs implement the ‘more efforts’ strategy is $U_{11}$:

$$U_{11}=yz\left(E_{1}\left({\bar{e}}_{1}\right)+R_{1}\left({\bar{e}}_{1}\right)-C_{1}\left({\bar{e}}_{1}\right)\right)+y\left(1-z\right)\left(R_{1}\left({\bar{e}}_{1}\right)-C_{1}\left({\bar{e}}_{1}\right)\right)+(1-\;y)z\left(E_{1}\left({\bar{e}}_{1}\right)+R_{1}\left({\bar{e}}_{1}\right)-C_{1}\left({\bar{e}}_{1}\right)\right)+\left(1-y\right)\left(1-z\right)\left(R_{1}\left({\bar{e}}_{1}\right)-C_{1}\left({\bar{e}}_{1}\right)\right).$$

The marginal expected revenue when HMIs implement the ‘less efforts’ strategy is $U_{12}$:

$$U_{12}=yz\left(E_{1}\left({\underline{e}}_{1}\right)+R_{1}\left({\underline{e}}_{1}\right)-C_{1}\left({\underline{e}}_{1}\right)\right)+y\left(1-z\right)\left(R_{1}\left({\underline{e}}_{1}\right)-C_{1}\left({\underline{e}}_{1}\right)\right)+\;\left(1-y\right)z\left(E_{1}\left({\underline{e}}_{1}\right)+R_{1}\left({\underline{e}}_{1}\right)-C_{1}\left({\underline{e}}_{1}\right)\right)+\left(1-y\right)\left(1-z\right)\left(R_{1}\left({\underline{e}}_{1}\right)-C_{1}\left({\underline{e}}_{1}\right)\right).$$

The expected revenue of the HMIs is $U_{1}$:

$$U_{1}=xU_{11}+\left(1-x\right)U_{12}=xzE_{1}\left({\bar{e}}_{1}\right)+\left(z-xz\right)E_{1}\left({\underline{e}}_{1}\right)\;+xR_{1}\left({\bar{e}}_{1}\right)-xC_{1}\left({\bar{e}}_{1}\right)+\left(1-x\right)R_{1}\left({\underline{e}}_{1}\right)-\left(1-x\right)C_{1}\left({\underline{e}}_{1}\right).$$

Thus, the replicator dynamics equation of the ‘efforts’ strategy chosen by HMIs can be written as F(x) in Formula (1):

(1) $$\begin{array}{cc} F\left(x\right)=dx/dt= & x\left(U_{11}-U_{1}\right)=x\left(1-x\right)(R_{1}\left({\bar{e}}_{1}\right)-C_{1}\left({\bar{e}}_{1}\right)-R_{1}\left({\underline{e}}_{1}\right)+C_{1}\left({\underline{e}}_{1}\right)+\\ zE_{1}\left({\bar{e}}_{1}\right)-zE_{1}\left({\underline{e}}_{1}\right)). & \end{array}$$

Likewise, the marginal expected revenues when PMIs implement the ‘more efforts’ and ‘less efforts’ strategies are $U_{21}$ and $U_{22}$, respectively:

$$U_{21}=xz\left(E_{2}\left({\bar{e}}_{2}\right)+R_{2}\left({\bar{e}}_{2}\right)-C_{2}\left({\bar{e}}_{2}\right)-\varepsilon B\right)+x\left(1-z\right)\left(R_{2}\left({\bar{e}}_{2}\right)-C_{2}\left({\bar{e}}_{2}\right)\right)+\;\left(1-x\right)z\left(E_{2}\left({\bar{e}}_{2}\right)+R_{1}\left({\bar{e}}_{2}\right)-C_{2}\left({\bar{e}}_{2}\right)-\varepsilon B\right)+\left(1-x\right)\left(1-z\right)\left(R_{2}\left({\bar{e}}_{2}\right)-C_{2}\left({\bar{e}}_{2}\right)\right),$$

$$U_{22}=xz\left(E_{2}\left({\underline{e}}_{2}\right)+R_{2}\left({\underline{e}}_{2}\right)-C_{2}\left({\underline{e}}_{2}\right)-\varepsilon B\right)+x\left(1-z\right)\left(R_{2}\left({\underline{e}}_{2}\right)-C_{2}\left({\underline{e}}_{2}\right)\right)+\;\left(1-x\right)z\left(E_{2}\left({\underline{e}}_{2}\right)+R_{2}\left({\underline{e}}_{2}\right)-C_{2}\left({\underline{e}}_{2}\right)-\varepsilon B\right)+\left(1-x\right)\left(1-z\right)\left(R_{2}\left({\underline{e}}_{2}\right)-C_{2}\left({\underline{e}}_{2}\right)\right).$$

The expected revenue of PMIs is $U_{2}$:

$$U_{2}=yU_{21}+\left(1-y\right)U_{22}=yzE_{2}\left({\bar{e}}_{2}\right)+\left(z-yz\right)E_{2}\left({\underline{e}}_{2}\right)+yR_{2}\left({\bar{e}}_{2}\right)-yC_{2}\left({\bar{e}}_{2}\right)+\;\left(1-y\right)R_{2}\left({\underline{e}}_{2}\right)-\left(1-y\right)C_{2}\left({\underline{e}}_{2}\right)-z\varepsilon B.$$

Then, the replicator dynamics equation of the ‘efforts’ strategy chosen by PMIs can be written as F(y) in Formula (2):

(2) $$\begin{array}{cc} F\left(y\right)=dy/dt= & y\left(U_{21}-U_{2}\right)=y\left(1-y\right)(R_{2}\left({\bar{e}}_{2}\right)-C_{2}\left({\bar{e}}_{2}\right)-R_{2}\left({\underline{e}}_{2}\right)+C_{2}\left({\underline{e}}_{2}\right)+\\ zE_{2}\left({\bar{e}}_{2}\right)-zE_{2}\left({\underline{e}}_{2}\right)). & \end{array}$$

Finally, the marginal expected revenues when patients carry out the ‘acceptance’ and ‘non-acceptance’ strategies are $U_{31}$ and $U_{32}$, respectively:

$$\begin{array}{ll} U_{31}= & xy\left(E_{3}\left({\bar{e}}_{2}\right)-\left(1-b\right)B\right)+x\left(1-y\right)\left(E_{3}\left({\underline{e}}_{2}\right)-\left(1-b\right)B-G\right)\\ & +\left(1-x\right)y\left(E_{3}\left({\bar{e}}_{2}\right)-\left(1-b\right)B-G\right)\\ & +\left(1-x\right)\left(1-y\right)\left(E_{3}\left({\underline{e}}_{2}\right)-\left(1-b\right)B-G\right) \end{array}$$

$$U_{32}=0$$

$$U_{3}=zU_{31}+\left(1-z\right)U_{32}=yzE_{3}\left({\bar{e}}_{2}\right)+z\left(1-y\right)E_{3}\left({\underline{e}}_{2}\right)+z\left(1-xy\right)G-z\left(1-b\right)B.$$

The replicator dynamics equation of the ‘acceptance’ strategy chosen by patients can be written as F(z) in Formula (3):

(3) $$\begin{array}{c} F\left(z\right)=dz/dt=z\left(U_{31}-U_{3}\right)=z\left(1-z\right)(yE_{3}\left({\bar{e}}_{2}\right)+\left(1-y\right)E_{3}\left({\underline{e}}_{2}\right)-\\ \left(1-xy\right)G-\left(1-b\right)B). \end{array}$$

### 5.2. Replicator Dynamic Analysis of the Tripartite Evolutionary Game

#### 5.2.1. Replicator Dynamic Analysis of the Patient Group

Formula (3) is the patient group’s replicator dynamic equation when they choose the ‘acceptance’ strategy. According to the stability theorem of differential equations and the property of evolutionary stability strategies (ESS), the stability point is required to satisfy the condition of $F^{\prime }\left(z\right)<0$.

When $x=x^{*}=-\frac{yE_{3}\left({\bar{e}}_{2}\right)+\left(1-y\right)E_{3}\left({\underline{e}}_{2}\right)-G-\left(1-b\right)B}{yG}$, then F(z)≡0. This means that no matter the value z takes, each z value is the replicator dynamics’ steady state.

When $x\neq x^{*}$, then F(z)=0, yielding z=0, z=1, both of which are fixed points and the steady states for the replicator dynamics of patients. The derivative of F(z) as regards z is $F^{\prime }\left(z\right)=\frac{\partial F\left(z\right)}{\partial z}=\left(1-2z\right)\left(yE_{3}\left({\bar{e}}_{2}\right)+\left(1-y\right)E_{3}\left({\underline{e}}_{2}\right)-\left(1-xy\right)G-\left(1-b\right)B\right)$, because yG>0(1>y >0). These two situations are discussed as follows:

(1) When $yE_{3}\left({\bar{e}}_{2}\right)+\left(1-y\right)E_{3}\left({\underline{e}}_{2}\right)-G-\left(1-b\right)B>0$, which means that the expected utility is more than the telemedicine fees and complaint costs, then $x^{*}<0$ is obtained. For every x that satisfies the condition 1>x >0, then $x>x^{*}$ is held. Based on the above, ${\frac{\partial F\left(z\right)}{\partial z}|}_{z=0}>0$ and ${\frac{\partial F\left(z\right)}{\partial z}|}_{z=1}<0$. Thus, z=1 is the steady state, and ‘acceptance’ the stable strategy.

(2) When $yE_{3}\left({\bar{e}}_{2}\right)+\left(1-y\right)E_{3}\left({\underline{e}}_{2}\right)-G-\left(1-b\right)B<0$, which means that the expected utility is less than the telemedicine fees and complaint costs, then $x^{*}>0$ is obtained. For every x that satisfies the condition 1>x >0, two situations result, discussed as follows:

① When $x>x^{*}$, then ${\frac{\partial F\left(z\right)}{\partial z}|}_{z=0}>0$ and ${\frac{\partial F\left(z\right)}{\partial z}|}_{z=1}<0$. Thus, z=1 is the steady state, and ‘acceptance’ the stable strategy.

② When $x<x^{*}$, then ${\frac{\partial F\left(z\right)}{\partial z}|}_{z=0}<0$ and ${\frac{\partial F\left(z\right)}{\partial z}|}_{z=1}>0$. Thus, z=0 is the steady state, and ‘non-acceptance’ the stable strategy.

**Figure 1:**
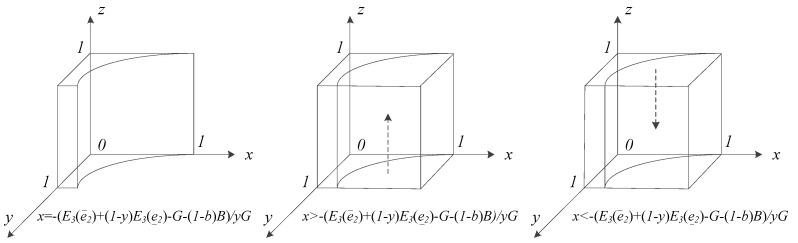
Dynamic evolution of patients’ evolutionarily stable strategy (ESS).

According to the above analysis, the dynamic evolutionary processes of patients’ ESS under various conditions are shown in Figure 1. In Figure 1, the *x*-axis, *y*-axis, and *z*-axis are stand for the value of *x*, *y*, and *z*, respectively. That is, the *x*-axis, *y*-axis, and *z*-axis represent the probability of adopting strategy of ‘HMI efforts’, ’PMI efforts’, and ’patients’ acceptance’, respectively. In Figure 1, the arrow points out to the direction of dynamic evolution of patients’ strategy. The box in Figure 1 indicates the probability of strategy space of three groups, HMIs, PMIs, and patients. For constraint threshold $x^{*}$, it is a function of y. Therefore, in the three-dimensional coordinate system, when we consider the variable z, others are paramenter.

Based on the analysis of the evolutionary game model, the following can be concluded:

Conclusion 1: Patients tend to choose the ‘acceptance’ strategy in telemedicine when (1) their expected utility or benefits are greater than the sum of the telemedicine fees and complaint costs, or (2) their expected utility or benefits are smaller than the sum of the telemedicine fees and complaint costs when x satisfies the condition $1>x>x^{*}$.

Conclusion 2: Patients tend to choose the ‘non-acceptance’ strategy in telemedicine when (1) their expected utility or benefits are less than the sum of the telemedicine fees and complaint costs or (2) their expected utility or benefits are greater than the sum of the telemedicine fees and complaint costs, when x satisfies the condition of $x^{*}>x>0$.

#### 5.2.2. Replicator Dynamic Analysis of PMIs

Formula (2) is the PMI group’s replicator dynamic equation when they choose the ‘efforts’ strategy. As per the above analysis, the necessary condition for ESS is $F^{\prime }\left(y\right)<0$.

When $z=z^{*}=\frac{R_{2}\left({\underline{e}}_{2}\right)-C_{2}\left({\underline{e}}_{2}\right)-R_{2}\left({\bar{e}}_{2}\right)+C_{2}\left({\bar{e}}_{2}\right)}{E_{2}\left({\bar{e}}_{2}\right)-E_{2}\left({\underline{e}}_{2}\right)}$, then F(y)≡0. This means that no matter the value y takes, each y value is the replicator dynamics’ steady state.

When $z\neq z^{*}$, then F(y)=0, yielding y=0, y=1. Both fixed points are the steady states for the replicator dynamics of PMIs. The derivative of F(y) regards y is $F^{\prime }\left(y\right)=\frac{\partial F\left(y\right)}{\partial y}=\left(1-2y\right)\left(R_{2}\left({\bar{e}}_{2}\right)-C_{2}\left({\bar{e}}_{2}\right)-R_{2}\left({\underline{e}}_{2}\right)+C_{2}\left({\underline{e}}_{2}\right)+zE_{2}\left({\bar{e}}_{2}\right)-zE_{2}\left({\underline{e}}_{2}\right)\right)$ because $E_{2}\left({\bar{e}}_{2}\right)-E_{2}\left({\underline{e}}_{2}\right)>0$. There are two situations to be discussed: 

(1) When $R_{2}\left({\underline{e}}_{2}\right)-C_{2}\left({\underline{e}}_{2}\right)<R_{2}\left({\bar{e}}_{2}\right)-C_{2}\left({\bar{e}}_{2}\right)$, which means that the net revenue under the ‘efforts’ strategy is more than that under the ‘no efforts’ strategy, for every z that satisfies the condition 1>z >0, then $z>z^{*}$. Based on the above, ${\frac{\partial F\left(y\right)}{\partial y}|}_{y=0}>0$ and ${\frac{\partial F\left(y\right)}{\partial y}|}_{y=1}<0$. Thus, y=1 is the steady state and ‘efforts’ the stable strategy.

(2) When $R_{2}\left({\underline{e}}_{2}\right)-C_{2}\left({\underline{e}}_{2}\right)>R_{2}\left({\bar{e}}_{2}\right)-C_{2}\left({\bar{e}}_{2}\right)$, which means that the net revenue under the ‘efforts’ strategy is less than that under the ‘no efforts’ strategy, then $z^{*}>0$ is obtained. For every z that satisfies the condition 1>z >0, there are two situations that result:

① When $z>z^{*}$, then ${\frac{\partial F\left(y\right)}{\partial y}|}_{y=0}>0$ and ${\frac{\partial F\left(y\right)}{\partial y}|}_{y=1}<0$. Thus, y=1 is the steady state, and ‘efforts’ the stable strategy.

② When $z<z^{*}$, then ${\frac{\partial F\left(y\right)}{\partial y}|}_{y=0}<0$ and ${\frac{\partial F\left(y\right)}{\partial y}|}_{y=1}>0$. Thus, y=0 is the steady state, and ‘no efforts’ the stable strategy.

**Figure 2:**
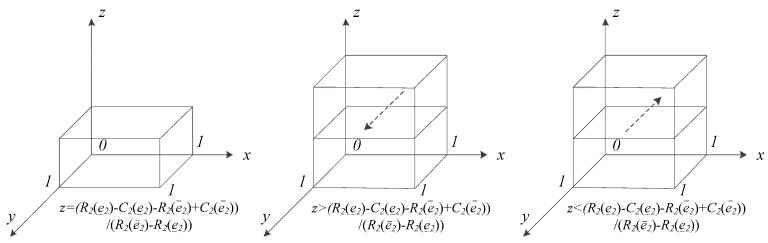
Dynamic evolution of ESS for PMIs.

According to the above analysis, the dynamic evolutionary processes of ESS of PMIs under various conditions are shown in Figure 2.

Based on the analysis of the evolutionary game model, the following can be concluded:

Conclusion 1: PMIs tend to choose the ‘efforts’ strategy in telemedicine when (1) the net revenue under the ‘efforts’ strategy is greater than that under the ‘no efforts’ strategy or (2) the net revenue under the ‘efforts’ strategy is less than that under the ‘no efforts’ strategy when z satisfies the condition $1>z>z^{*}$.

Conclusion 2: PMIs tend to choose the ‘no efforts’ strategy in telemedicine, when (1) the net revenue under the ‘efforts’ strategy is less than that under the ‘no efforts’ strategy or (2) the net revenue under the ‘efforts’ strategy is greater than that under the ‘no efforts’ strategy, when z satisfies the condition $0<z<z^{*}$.

#### 5.2.3. Replicator Dynamics Analysis of HMIs

Formula (1) is the HMI group’s replicator dynamics equation when they choose the ‘efforts’ strategy. As per the above analysis, the necessary condition for ESS is $F^{\prime }\left(x\right)<0$.

When $z=z^{\prime }=\frac{R_{1}\left({\underline{e}}_{1}\right)-C_{1}\left({\underline{e}}_{1}\right)-R_{1}\left({\bar{e}}_{1}\right)+C_{1}\left({\bar{e}}_{1}\right)}{E_{1}\left({\bar{e}}_{1}\right)-E_{1}\left({\underline{e}}_{1}\right)}$, then F(x)≡0. This means that no matter the value that x takes, each x value is the replicator dynamics’ steady state.

When $z\neq z^{\prime }$, then F(x)=0, yielding x=0, x=1, both of which are fixed points and the steady states for the replicator dynamics of HMIs. The derivative of F(x) as regards x is $F^{\prime }\left(x\right)=\frac{\partial F\left(x\right)}{\partial x}=\left(1-2x\right)\left(R_{1}\left({\bar{e}}_{1}\right)-C_{1}\left({\bar{e}}_{1}\right)-R_{1}\left({\underline{e}}_{1}\right)+C_{1}\left({\underline{e}}_{1}\right)+zE_{1}\left({\bar{e}}_{1}\right)-zE_{1}\left({\underline{e}}_{1}\right)\right)$ because $E_{1}\left({\bar{e}}_{1}\right)-E_{1}\left({\underline{e}}_{1}\right)>0$. There are two situations to discuss:

(1) When $R_{1}\left({\underline{e}}_{1}\right)-C_{1}\left({\underline{e}}_{1}\right)<R_{1}\left({\bar{e}}_{1}\right)-C_{1}\left({\bar{e}}_{1}\right)$, which means that the net revenue under the ‘efforts’ strategy is greater than that under the ‘no efforts’ strategy, for every z that satisfies the condition 1>z >0, then $z>z^{\prime }$. Based on the above, ${\frac{\partial F\left(x\right)}{\partial x}|}_{x=0}>0$ and ${\frac{\partial F\left(x\right)}{\partial x}|}_{x=1}<0$. Thus, x=1 is the steady state and ‘efforts’ the stable strategy.

(2) When $R_{1}\left({\underline{e}}_{1}\right)-C_{1}\left({\underline{e}}_{1}\right)>R_{1}\left({\bar{e}}_{1}\right)-C_{1}\left({\bar{e}}_{1}\right)$, which means that the net revenue under the ‘efforts’ strategy is less than that under the ‘no efforts’ strategy, then $z^{\prime }>0$ is obtained. For every z which satisfies the condition 1>z >0, there are two situations that require discussion:

① When $z>z^{\prime }$, ${\frac{\partial F\left(x\right)}{\partial x}|}_{x=0}>0$ and ${\frac{\partial F\left(x\right)}{\partial x}|}_{x=1}<0$. Thus, x=1 is the steady state, and ‘efforts’ the stable strategy.

② When $z<z^{\prime }$, then ${\frac{\partial F\left(x\right)}{\partial x}|}_{x=0}<0$ and ${\frac{\partial F\left(x\right)}{\partial x}|}_{x=1}>0$. Thus, x=0 is the steady state, and ‘no efforts’ the stable strategy.

**Figure 3:**
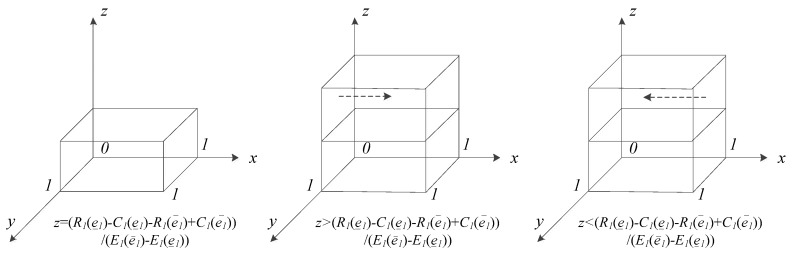
Dynamic evolution of ESS of HMIs.

According to the above analysis, the dynamic evolutionary processes of ESS of HMIs under various conditions are shown in Figure 3.

Based on the analysis of the evolutionary game model, the following can be concluded:

Conclusion 1: HMIs tend to choose the ‘efforts’ strategy in telemedicine when (1) the net revenue under the ‘efforts’ strategy is greater than that under the ‘no efforts’ strategy or (2) the net revenue under the ‘efforts’ strategy is less than that under the ‘no efforts’ strategy, when z satisfies the condition $1>z>z^{\prime }$.

Conclusion 2: HMIs tend to choose the ‘no efforts’ strategy in telemedicine when (1) the net revenue under the ‘efforts’ strategy is less than that under the ‘no efforts’ strategy or (2) the net revenue under the ‘efforts’ strategy is greater than that under the ‘no efforts’ strategy when z satisfies the condition $0<z<z^{\prime }$.

### 5.3. Stability Analysis of the Local Equilibrium Points (EPs)

According to the above three replicator equations, F(x), F(y), and F(z), a three-dimensional dynamic evolutionary system of HMIs, PMIs, and patients can be obtained, as in Equation (4).

(4) $$\left\{ \begin{array}{l} F\left(x\right)=x\left(1-x\right)\left(R_{1}\left({\bar{e}}_{1}\right)-C_{1}\left({\bar{e}}_{1}\right)-R_{1}\left({\underline{e}}_{1}\right)+C_{1}\left({\underline{e}}_{1}\right)+zE_{1}\left({\bar{e}}_{1}\right)-zE_{1}\left({\underline{e}}_{1}\right)\right)\\ F\left(y\right)=y\left(1-y\right)\left(R_{2}\left({\bar{e}}_{2}\right)-C_{2}\left({\bar{e}}_{2}\right)-R_{2}\left({\underline{e}}_{2}\right)+C_{2}\left({\underline{e}}_{2}\right)+zE_{2}\left({\bar{e}}_{2}\right)-zE_{2}\left({\underline{e}}_{2}\right)\right)\\ F\left(z\right)=z\left(1-z\right)\left(yE_{3}\left({\bar{e}}_{2}\right)+\left(1-y\right)E_{3}\left({\underline{e}}_{2}\right)-\left(1-xy\right)G-\left(1-b\right)B\right) \end{array}\right.$$

According to the method introduced by Friedman [30], the local stability of the *Jacobian matrix* system can be used to determine the evolutionary stability strategies of the dynamic equation set. The *Jacobian matrix* of the replication dynamic Equation (4) is shown below:

$$J=\left[\begin{array}{ccc} \partial F\left(x\right)∕\partial x & \partial F\left(x\right)∕\partial y & \partial F\left(x\right)∕\partial z\\ \partial F\left(y\right)∕\partial x & \partial F\left(y\right)∕\partial y & \partial F\left(y\right)∕\partial z\\ \partial F\left(z\right)∕\partial x & \partial F\left(z\right)∕\partial y & \partial F\left(z\right)∕\partial z \end{array}\right]=$$

(5) $$[\begin{array}{ccc} \begin{array}{c} \left(1-2x\right)(R_{1}\left({\bar{e}}_{1}\right)-C_{1}\left({\bar{e}}_{1}\right)-R_{1}\left({\underline{e}}_{1}\right)\\ +C_{1}\left({\underline{e}}_{1}\right)+zE_{1}\left({\bar{e}}_{1}\right)-zE_{1}\left({\underline{e}}_{1}\right)) \end{array} & 0 & x\left(1-x\right)\left(E_{1}\left({\bar{e}}_{1}\right)-E_{1}\left({\underline{e}}_{1}\right)\right)\\ 0 & \begin{array}{c} \left(1-2y\right)(R_{2}\left({\bar{e}}_{2}\right)-C_{2}\left({\bar{e}}_{2}\right)-R_{2}\left({\underline{e}}_{2}\right)\\ +C_{2}\left({\underline{e}}_{2}\right)+zE_{2}\left({\bar{e}}_{2}\right)-zE_{2}\left({\underline{e}}_{2}\right)) \end{array} & y\left(1-y\right)\left(E_{2}\left({\bar{e}}_{2}\right)-E_{2}\left({\underline{e}}_{2}\right)\right)\\ z\left(1-z\right)yG & z\left(1-z\right)\left(E_{3}\left({\bar{e}}_{2}\right)-E_{3}\left({\underline{e}}_{2}\right)+xG\right) & \begin{array}{c} \left(1-2z\right)(yE_{3}\left({\bar{e}}_{2}\right)-\left(1-b\right)B\\ +\left(1-y\right)E_{3}\left({\underline{e}}_{2}\right)-\left(1-xy\right)G) \end{array} \end{array}].$$

In the system (4), let F(x)=F(y)=F(z)=0, whereby nine local EPs can be obtained, respectively: $D_{1}\left(0,0,0\right),\;D_{2}\left(0,0,1\right),\;D_{3}\left(0,1,0\right),\;D_{4}\left(0,1,1\right),\;D_{5}\left(1,0,0\right),\;D_{6}\left(1,0,1\right),\;D_{7}\left(1,1,0\right),\;D_{8}\left(1,1,1\right),\;D_{9}\left(x_{0},y_{0},z_{0}\right)$. According to the research of Selten and Ritzberger [31], only if the strategy combination is a strict Nash equilibrium (NE) will it be asymptotically stable in the replicator dynamic system of the multi-group evolutionary game; which is to say that if the evolutionary game equilibrium strategy combination is asymptotically stable, then this strategy combination must be a strict NE, and the strict NE a pure strategy NE (PSNE). Thus, for the replicator dynamics among HMIs, PMIs, and patients, only the first eight EPs need to be explored. From an analysis of the local EP $D_{1}\left(0,0,0\right)$ and its Jacobian matrix, the following can be shown:

$$\left[\begin{array}{ccc} R_{1}\left({\bar{e}}_{1}\right)-C_{1}\left({\bar{e}}_{1}\right)-R_{1}\left({\underline{e}}_{1}\right)+C_{1}\left({\underline{e}}_{1}\right) & 0 & 0\\ 0 & R_{2}\left({\bar{e}}_{2}\right)-C_{2}\left({\bar{e}}_{2}\right)-R_{2}\left({\underline{e}}_{2}\right)+C_{2}\left({\underline{e}}_{2}\right) & 0\\ 0 & 0 & E_{3}\left({\underline{e}}_{2}\right)-\left(1-b\right)B-G \end{array}\right]$$

It is clear that the eigenvalues of the above Jacobian matrix are $\lambda_{1}=R_{1}\left({\bar{e}}_{1}\right)-C_{1}\left({\bar{e}}_{1}\right)-R_{1}\left({\underline{e}}_{1}\right)+C_{1}\left({\underline{e}}_{1}\right),\;\lambda_{2}=R_{2}\left({\bar{e}}_{2}\right)-C_{2}\left({\bar{e}}_{2}\right)-R_{2}\left({\underline{e}}_{2}\right)+C_{2}\left({\underline{e}}_{2}\right)+E_{2}\left({\bar{e}}_{2}\right)-E_{2}\left({\underline{e}}_{2}\right),\;\lambda_{3}=E_{3}\left({\underline{e}}_{2}\right)-\left(1-b\right)B-G$, respectively. Likewise, all eigenvalues for the other seven EPs can be obtained when these EPs are replaced in the Jacobian matrix. This is expressed by Formula (4). All eigenvalues of each EP’s Jacobian matrix are shown in Table 2.

According to research by Friedman [30], the judgement criterion of EP’s stability can be summarized as follows: When the symbol of each eigenvalue is negative, then the evolution EP is a stable point; when the symbol of each eigenvalue is positive, then the evolution equilibrium is an unstable point; when the eigenvalues are neither all negative nor positive at the same time, then the evolution equilibrium is a saddle point.

The effect or performance of telemedicine is mainly influenced by the behavior of HMIs, PMIs, and patients, and the key factors that influence these three groups are the relationships between the costs and benefits of their behavioral strategies. To simplify the analysis of the eigenvalue symbols that correspond to different EPs, in general there are two situations to discuss: (1) When the costs are greater than the benefits for all three groups, three conditions should be met, namely $C_{1}\left({\bar{e}}_{1}\right)-C_{1}\left({\underline{e}}_{1}\right)>\left(E_{1}\left({\bar{e}}_{1}\right)+R_{1}\left({\bar{e}}_{1}\right)\right)-\left(E_{1}\left({\underline{e}}_{1}\right)+R_{1}\left({\underline{e}}_{1}\right)\right),\;C_{2}\left({\bar{e}}_{2}\right)-C_{2}\left({\underline{e}}_{2}\right)>\left(E_{2}\left({\bar{e}}_{2}\right)+R_{2}\left({\bar{e}}_{2}\right)\right)-\left(E_{2}\left({\underline{e}}_{2}\right)+R_{2}\left({\underline{e}}_{2}\right)\right)$, and $\left(1-b\right)B>E_{3}\left({\bar{e}}_{2}\right)$; (2) when the costs are less than the benefits for all three groups, three conditions should also be met, namely $C_{1}\left({\bar{e}}_{1}\right)-C_{1}\left({\underline{e}}_{1}\right)<\left(E_{1}\left({\bar{e}}_{1}\right)+R_{1}\left({\bar{e}}_{1}\right)\right)-\left(E_{1}\left({\underline{e}}_{1}\right)+R_{1}\left({\underline{e}}_{1}\right)\right)$, $C_{2}\left({\bar{e}}_{2}\right)-C_{2}\left({\underline{e}}_{2}\right)<\left(E_{2}\left({\bar{e}}_{2}\right)+R_{2}\left({\bar{e}}_{2}\right)\right)-\left(E_{2}\left({\underline{e}}_{2}\right)+R_{2}\left({\underline{e}}_{2}\right)\right)$, and $\left(1-b\right)B+G<E_{3}\left({\underline{e}}_{2}\right)$. Table 3 shows the eigenvalue symbols that correspond to different EPs under the aforementioned two situations.

By analyzing the local stability of the *Jacobian matrix*, in situation 1, point $D_{1}\left(0,0,0\right)$ is the evolution stabilization point, point $D_{8}\left(1,1,1\right)$ is the unstable point, and the other six points make up the saddle point. This means that the tripartite dynamic system will gradually converge on point $D_{1}\left(0,0,0\right)$, which is to say that when the HMIs and PMIs choose the ‘no efforts’ strategy, patients choose the ‘non-acceptance’ strategy. Thus, when the costs are greater than the benefits, the evolutionary behavior of the three groups will hinder the promotion of telemedicine.

Likewise, in situation 2, point $D_{8}\left(1,1,1\right)$ is the evolution stabilisation point, point $D_{0}\left(0,0,0\right)$ is the unstable point, and the other six points make up the saddle point. This means that the tripartite dynamic system will gradually converge on point $D_{8}\left(1,1,1\right)$. In other words, when the HMIs and PMIs choose the ‘efforts’ strategy, patients choose the ‘acceptance’ strategy. Thus, when the costs are less than the benefits, the evolutionary behaviors of the three groups will improve the promotion of telemedicine. This evolutionary state is ideal given the policy objective of implementing telemedicine in China. Therefore, the government should take measures to increase the benefits and decrease the costs of using telemedicine across all three groups. This includes increasing the financial support for MIs to provide telemedicine, promoting payment reforms to reflect the efforts MIs are making to provide telemedicine, decreasing telemedicine fees and complaint costs, and improving the social medical insurance reimbursement system. These measures will promote telemedicine and optimize the allocation of limited health resources.

## 6. Simulation Analysis

To more intuitively show the evolutionary process of the behavior/strategy of the players (patients, PMIs, and HMIs) and to verify the fitness of the tripartite evolutionary game model constructed above, Matlab was used to simulate the evolutionary process and interactivity of HMIs, PMIs, and patients. It was also used to analyze the conditions under which the tripartite evolutionary game’s ESS takes less time, given the parameters in situation 1. Lastly, it yielded policy implications for the promotion of telemedicine in China.

**Figure 4:**
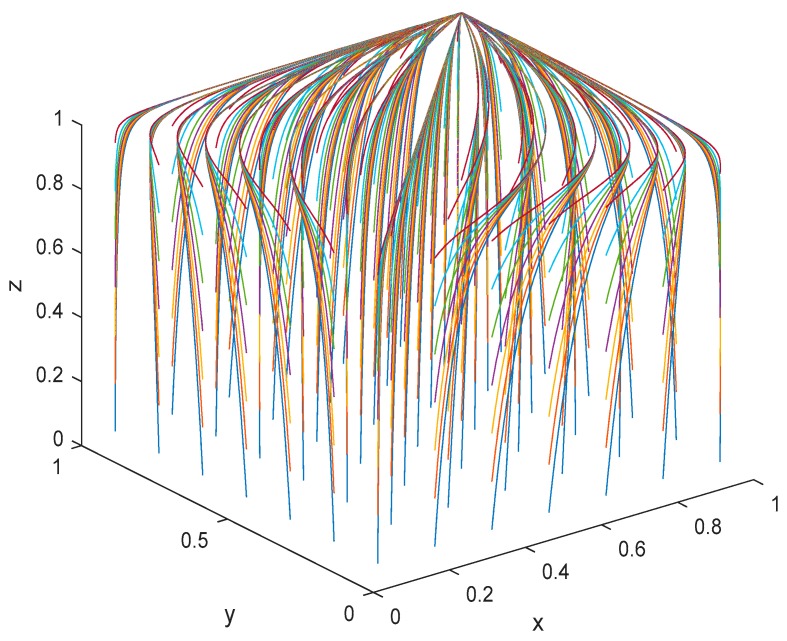
Three-dimensional evolutionary game for EP $D_{8}\left(1,1,1\right)$.

When the costs are less than the benefits for all three groups, the EP is $D_{8}\left(1,1,1\right)$ under the conditions $C_{1}\left({\bar{e}}_{1}\right)-C_{1}\left({\underline{e}}_{1}\right)<\left(E_{1}\left({\bar{e}}_{1}\right)+R_{1}\left({\bar{e}}_{1}\right)\right)-\left(E_{1}\left({\underline{e}}_{1}\right)+R_{1}\left({\underline{e}}_{1}\right)\right)$, $C_{2}\left({\bar{e}}_{2}\right)-C_{2}\left({\underline{e}}_{2}\right)<\left(E_{2}\left({\bar{e}}_{2}\right)+R_{2}\left({\bar{e}}_{2}\right)\right)-\left(E_{2}\left({\underline{e}}_{2}\right)+R_{2}\left({\underline{e}}_{2}\right)\right)$, and $\left(1-b\right)B+G<E_{3}\left({\underline{e}}_{2}\right)$. In this paper, it was assumed that the initial time of simulation was 0 and the end time was 20. For the parameters in the model, it was assumed that $R_{1}\left({\bar{e}}_{1}\right)=4,\;R_{1}\left({\underline{e}}_{1}\right)=3,\;R_{2}\left({\bar{e}}_{2}\right)=2,\;R_{2}\left({\underline{e}}_{2}\right)=1,\;C_{1}\left({\bar{e}}_{1}\right)=4$, ${\;C}_{1}\left({\underline{e}}_{1}\right)=3,\;C_{2}\left({\bar{e}}_{2}\right)=2$, ${\;C}_{2}\left({\underline{e}}_{2}\right)=1,\;E_{1}\left({\bar{e}}_{1}\right)=5,\;E_{1}\left({\underline{e}}_{1}\right)=4,\;E_{2}\left({\bar{e}}_{2}\right)=3,\;E_{2}\left({\underline{e}}_{2}\right)=2,\;E_{3}\left({\bar{e}}_{2}\right)=5,\;E_{3}\left({\underline{e}}_{2}\right)=4,\;G=1,\;B=2,\;\;\mathrm{and}\;b=0.7$. In addition, it was assumed that the initial intention of the ‘efforts’ strategy chosen by HMIs and PMIs ranged from 0.05 to 0.95, and that the time interval was 0.15. Likewise, it was assumed that the initial intention of the ‘acceptance’ strategy chosen by patients ranged from 0.05 to 0.95, with 0.15 as the time interval. The three-dimensional simulation results were then obtained, as shown in Figure 4. It is clear that all the curves converge on EP $D_{8}\left(1,1,1\right)$, which verifies the analysis of the above EPs and confirms the dynamic evolution results. This means that the strategy combination (‘HMI efforts’, ‘PMI efforts’, ‘patients’ acceptance’) is ESS when the costs are less than the benefits of all three groups’ behaviors. Thus, if the government can increase the benefits and decrease the costs for MIs and patients either through policy tools or technological innovation, the three-dimensional dynamic system will evolve to EP $D_{8}\left(1,1,1\right)$, which will stimulate the promotion of telemedicine in China. For EP $D_{8}\left(1,1,1\right)$, the HMIs and PMIs both chose the ‘efforts’ strategy for providing telemedicine, while the patients chose the ‘acceptance’ strategy. The positive interaction of these three groups not only promotes telemedicine in China, but also optimizes the foundation of the hierarchical medical system.

### 6.1. Change in the Initial Intention of the Strategy Combination (‘HMI Efforts’, ‘PMI Efforts’, ‘Patients’ Acceptance’)

#### 6.1.1. Scenario x<y<z

**Figure 5:**
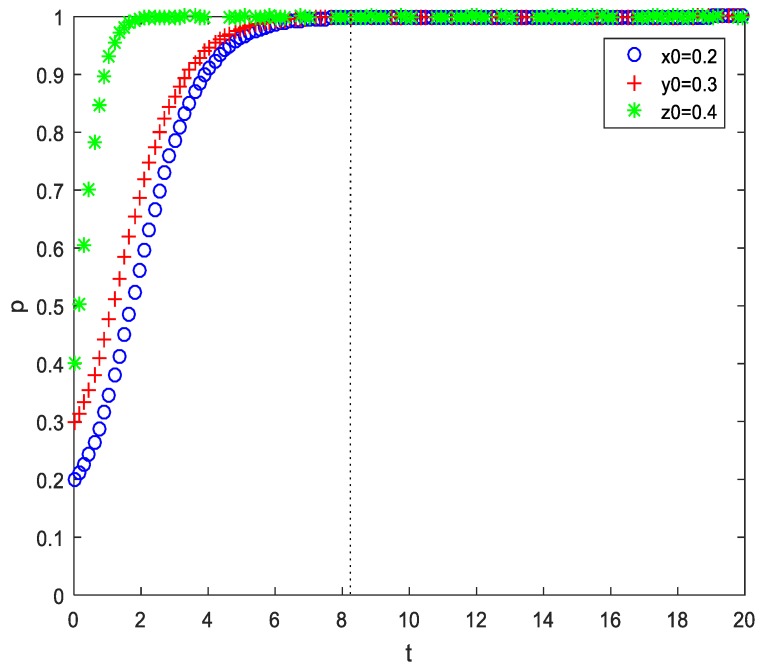
Time series of EP $D_{8}\left(1,1,1\right)$ (initial value (0.2,0.3,0.4), b=0.7).

**Figure 6:**
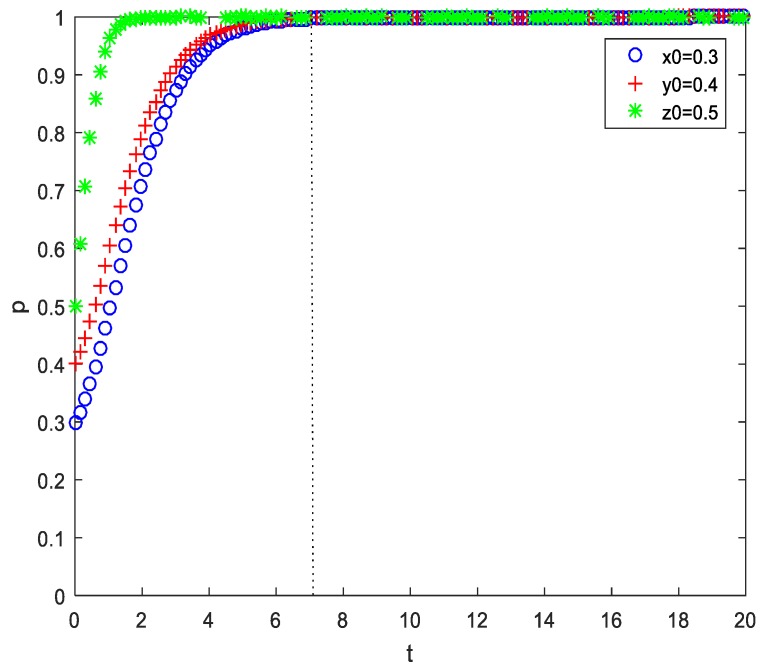
Time series of EP $D_{8}\left(1,1,1\right)$ (original value (0.3,0.4,0.5), b=0.7).

In the first scenario, the initial intention of patients’ ‘acceptance’ strategy was higher than both that of PMIs’ ‘efforts’ strategy and HMIs’ ‘efforts’ strategy. Apart from the parameter assigned above, only an initial value was assigned here of (x,y,z) as (0.2,0.3,0.4) and (0.3,0.4,0.5), respectively. The results of the numerical simulation are shown in Figure 5 and Figure 6. Comparing these two figures, the initial intention of the strategy combination (‘efforts’, ‘efforts’, ‘acceptance’) was (0.2,0.3,0.4) and ESS was achieved at t=8.55. However, when the initial intention of the strategy combination was increased by 0.1 to (0.3,0.4,0.5), ESS was achieved at t=7.80. Furthermore, we also simulated the situations of (0.4,0.5,0.6), (0.5,0.6,0.7), (0.6,0.7,0.8), and (0.7,0.8,0.9), which respectively achieved ESS at t=7.65, t=6.90, t=6.15, and t=5.85. This result shows that the higher the initial intention of the strategy combination (‘HMI efforts’, ‘PMI efforts’, ‘patiens’ acceptance’), the less time it takes for ESS to be achieved. In addition, if x<y<z, regardless of the values they hold, the evolutionary time of patients, PMIs, and HMIs choosing (‘efforts’, ‘efforts’, or ‘acceptance’) increase in turn. This shows that in the process of promoting telemedicine, patients’ behavior is not the largest obstacle compared to the MIs. At the same time, it also shows that in the process of implementing telemedicine, government information should be in place so that patients can understand the new telemedicine services and more easily accept them. Moreover, patients can enjoy the same high quality of service provided by HMIs without travelling long distances, which cuts their costs and increases their benefits. However, in contrast, the evolutionary time of PMIs and HMIs lags behind that of patients. Thus, the government should focus more on HMIs and PMIs in the process of promoting telemedicine, using various incentive and benefits mechanisms. This would enhance the quality of MIs, ensuring they provide top-rate telemedicine services.

#### 6.1.2. Scenario x>y>z

In the second scenario, the initial intention of patients’ ‘acceptance’ strategy is less than that of both PMIs’ ‘efforts’ strategy and HMIs’ ‘efforts’ strategy. Apart from the parameter assigned at the beginning, only an initial value of (x,y,z) was assigned here as (0.4,0.3,0.2) and (0.5,0.4,0.3), respectively. Comparing these two numerical simulation situations, when the initial intention of the strategy combination (‘HMI efforts’, ‘PMI efforts’, ‘patients’ acceptance’) was (0.4,0.3,0.2), ESS was achieved at t=8.1. However, when the initial intention of the strategy combination was increased by 0.1 to (0.5,0.4,0.3), ESS was achieved at t=7.50. Similarly, we get t=7.05, t=6.60, t=5.85, and t=5.10, if we continue to increase the initial probability of the strategy combination to (0.6,0.5,0.4), (0.7,0.6,0.5), (0.8,0.7,0.6), and (0.9,0.8,0.7), respectively. This result shows that the higher the initial intention of the strategy combination (‘HMI efforts’, ‘PMI efforts’, ‘patients’ acceptance’), the less time it takes to achieve ESS. In addition, if x>y>z, regardless of the values they hold, the evolutionary time of patients, HMIs, and PMIs in choosing (‘efforts’, ‘efforts’, or ‘acceptance’) increases. Combined with the above analysis, whether the initial intention of the ‘acceptance’ strategy chosen by patients is highest or lowest among the three groups, the results are that the patients reach the steady state in the evolutionary game in the shortest time. Therefore, if the government can take measures to shorten the evolution time of MIs, this will help the promotion of telemedicine.

#### 6.1.3. Scenario x=y=z

In the third scenario, the initial intention of patients’ ‘acceptance’ strategy is the same as that of PMIs’ ‘efforts’ strategy and HMIs’ ‘efforts’ strategy. Apart from the parameter assigned at the beginning, only an initial value of (x,y,z) was assigned here as (0.2,0.2,0.2) and (0.3,0.3,0.3), respectively. Comparing these two simulation situations, when the initial intention of the strategy combination (‘HMI efforts’, ‘PMI efforts’, ‘patients’ acceptance’) was (0.2,0.2,0.2), ESS was achieved at t=8.40. However, when the initial intention of the strategy combination was increased by 0.1 to (0.3,0.3,0.3), ESS was achieved at t=7.95. Similarly, we get t=7.65, t=7.50, t=7.35, t=6.90, t=6.15, and t=5.70 if we continue to increase the initial probability of the strategy combination to (0.4,0.4,0.4), (0.5,0.5,0.5), (0.6,0.6,0.6), (0.7,0.7,0.7), (0.8,0.8,0.8), and (0.9,0.9,0.9), respectively. This shows that the higher the initial intention of the strategy combination (‘HMI efforts’, ‘PMI efforts’, ‘patients’ acceptance’), the less time it takes to achieve ESS. In addition, if x=y=z, regardless the values they hold, the evolutionary time of patients in choosing the ‘acceptance’ strategy is the shortest of all three groups, which is consistent with the above reported results. In addition, HMIs and PMIs show the same evolutionary time in choosing the ‘efforts’ strategy, revealing a time lag behind that of the patient group. Therefore, even if the initial intention of the three parties is the same, the patient group will reach the steady state in the evolutionary game in the shortest time.

### 6.2. Change in Telemedicine Fees for Patients

To verify whether a reduction in telemedicine fees further promotes telemedicine and has a positive impact on all the groups involved, with other parameters unchanged, B=2 was reduced to B=1; moreover, the initial value for choosing the (‘HMI efforts’, ‘PMI efforts’, or ‘patients’ acceptance’) strategy was given as increasing trend from (0.2,0.3,0.4) to (0.7,0.8,0.9), decreasing trend from (0.4,0.3,0.2) to (0.9,0.8,0.7), and same value from (0.2,0.2,0.2) to (0.9,0.9,0.9), respectively. The results of the numerical simulation can be summarized as follows:

**(1) The evolution results of the three groups with reduced telemedicine fees (Scenario**x<y<z**).** By comparing all situations that were assumed before and after changing telemedicine fees (B), it can be concluded that before the telemedicine fees were reduced, ESS of all groups was achieved at t=8.55, t=7.80, t=7.65, t=6.90, t=6.15, and t=5.85 of the six situations, respectively. However, after reducing the telemedicine fees, ESS of all groups was achieved to t=8.47, t=7.35, t=7.05, t=6.45, t=6.00, and t=5.40 of the six situations, respectively. Thus, it is clear that reducing patients’ telemedicine fees can shorten the evolutionary time. This means that reducing telemedicine fees is beneficial for promoting telemedicine. Such a result has important policy implications, namely that the government should focus on the pricing of telemedicine and reducing fees through technological innovation or other policy measures. 

**(2) The evolution results of the three groups with reduced telemedicine fees (Scenario**x>y>z**).** Before reducing the telemedicine fees, ESS of all groups was estimated at t=8.1, t=7.80, t=7.65, t=6.90, t=6.15, and t=5.85 of the six situations, respectively. However, after reducing the telemedicine fees, ESS of all groups was estimated at t=7.65, t=6.95, t=6.90, t=6.67, t=6.00, and t=5.55 of the six situations respectively. Therefore, it is obvious that reducing patients’ telemedicine fees can shorten the evolutionary time, implying that a reduction in telemedicine fees can be a means for promoting telemedicine. 

**(3) The evolution results of the three groups with reduced telemedicine fees (Scenario**x=y=z)**.** Before the telemedicine fees were reduced, ESS of all groups was achieved at t=8.4, t=7.95, t=7.65, t=7.50, t=7.35, t=6.90, t=6.15, and t=5.70 of the eight situations, respectively. However, after reducing the telemedicine fees, ESS of all groups was achieved at t=8.02, t=7.88, t=7.20, t=7.12, t=6.92, t=6.30, t=5.85, and t=5.25 of the eight situations, respectively. Thus, reducing patients’ telemedicine fees can shorten the evolutionary time. 

Together, these results suggest that after reducing telemedicine fees and regardless the initial intention of HMIs, PMIs, and patients in choosing the strategy combination (‘efforts’, ‘efforts’, ‘acceptance’), the time required for the three parties to evolve to ESS was significantly shortened, indicating that a reduction in telemedicine fees can effectively promote the telemedicine system.

### 6.3. Change in the Reimbursement Ratio of Telemedicine Fees

#### 6.3.1. Telemedicine Services Are Embedded in Social Medical Insurance Reimbursements

(1) Scenario x<y<z. To simplify the analysis, it was assumed that the two reimbursement ratio standards for telemedicine fees were 0.9 and 0.3, respectively. The former was the highest reimbursement ratio of Chinese medical insurance in very poor areas, while the latter was the lowest reimbursement ratio of Chinese medical insurance for non-referral patients in rural areas. In addition, to discuss the optimal range of the reimbursement ratio, 0.4, 0.5, 0.6, 0.7, and 0.8 were chosen as reimbursement ratio standards. The initial intention of the strategy combination (‘HMI efforts’, ‘PMI efforts’, ‘patients’ acceptance’) was (0.2,0.3,0.4), but when parameter b was modified, the results were t=8.70, t=8.98, t=8.85, t=8.91, t=8.55, t=8.63, and t=8.85, respectively, when b=0.3~0.9, with 0.1 as interval across the three strategies. Thus, compared with the initial parameter value b=0.7, increasing (b=0.9, or b=0.8) or decreasing (b=0.3~0.6, with 0.3 as interval) the reimbursement ratio influences the evolutionary results of the three groups. Compared with situation of different b value, when the reimbursement ratio increases (decreases), the evolutionary time required for ESS increases slightly, showing that the optimal reimbursement ratio of medical insurance is neither the higher one nor the lower one. Combined with the above-mentioned results, the reimbursement ratio between 0.7 and 0.8 in our model is the appropriate ratio.

(2) Scenario x>y>z. In this scenario, 0.3~0.6 and 0.8~0.9 both with 0.1 as interval were chosen as the reimbursement ratios, compared with the scenario in which 0.7 was the reimbursement ratio. Here, (0.4,0.3,0.2) was the initial intention of the strategy combination (‘HMI efforts’, ‘PMI efforts’, ‘patients’ acceptance’); and we can obtain a total of seven situations when the value b changes. A look at the ESS time of these seven situations shows that the results are the same as in scenario x<y<z, i.e., the optimal reimbursement ratio range is 0.7–0.8.

(3) Scenario x=y=z. In this scenario, 0.3~0.6, and 0.8~0.9 with 0.1 as interval were chosen as the reimbursement ratios, compared with the scenario in which 0.7 was the reimbursement ratio. Here, (0.2,0.2,0.2) was the initial intention of the strategy combination (‘HMI efforts’, ‘PMI efforts’, ‘patients’ acceptance’). The seven results of ESS time are obtained by means of the numerical simulation. By analyzing these evolutionary time series of the three parties, the results were found to be the same as in scenarios x<y<z and x>y>z, i.e., the optimal reimbursement ratio range is still 0.7–0.8.

In summary, after incorporating telemedicine fees into the medical insurance reimbursement scheme, based on numerical simulation, the optimal reimbursement ratio for promoting telemedicine is in the range of 0.7–0.8.

#### 6.3.2. Telemedicine Services Are Not Embedded in Medical Insurance Reimbursements

In China, some provinces or cities explicitly stipulate that telemedicine services are embedded in medical insurance reimbursements while others do not. Therefore, a discussion was required about the situation when telemedicine services are not embedded in such reimbursements. In Section 6.3.1, the reimbursement ratio of telemedicine fees is zero. The initial intention of strategy combination (‘HMI efforts’, ‘PMI efforts’, ‘patients’ acceptance’) remained (0.2,0.3,0.4), but b=0.7 was changed to b=0 in this simulation. This means that telemedicine services are not embedded in medical insurance reimbursement, which is common in some areas in China. Therefore, the situation in this case also has policy implications. There are still three situations with considerable differences in the initial intention of strategy combination (‘HMI efforts’, ‘PMI efforts’, ‘patients’ acceptance’).

The first is scenario x<y<z. Comparing evolutionary time of b=0 with that of b=0.3~0.9, when b=0, telemedicine services are not embedded in social medical insurance reimbursement; moreover, the strategy combination (‘efforts’, ‘efforts’, ‘acceptance’) chosen by HMIs, PMIs and patients respectively will only reach ESS at t=10.05. Comparing evolutionary times at t=8.55~8.98 (when b=0.3~0.9), it can be concluded that telemedicine services are embedded in medical insurance reimbursements, which is beneficial for shortening the evolutionary time of ESS.

The second scenario is x>y>z. Comparing evolutionary time of b=0 with that of b=0.3~0.9, there is a similar conclusion to that of situation x<y<z, i.e., with differing initial intentions regards the strategy combination (‘HMI efforts’, ‘PMI efforts’, ‘patients’ acceptance’), telemedicine services that are not embedded in medical insurance reimbursements will hinder the promotion of telemedicine.

Finally, assuming that the initial intentions of the strategy combination (‘HMI efforts’, ‘PMI efforts’, ‘patients’ acceptance’) are the same, the impact of parameter b=0 on the evolutionary time of ESS was discussed. Comparing the situation when of b=0 with that of when b=0.3~0.9, we found that telemedicine fees that are not embedded in medical insurance reimbursement hinder the promotion of telemedicine. Considering the optimal reimbursement ratio (0.7–0.8), the impact of telemedicine fees not embedded in medical insurance reimbursement on evolutionary times was not significant compared with the highest or lowest reimbursement ratio (highest being 0.9, lowest being 0.3).

In summary, regardless of the initial intention, the conclusions are consistent in finding that telemedicine services that are not embedded in medical insurance reimbursement will hinder the promotion of such services.

## 7. The Telemedicine Service of Guizhou Province

### 7.1. The Price Reform of Telemedicine in Guizhou

Since 2014, telemedicine has been developing rapidly in Guizhou province. The province was the first in China that has successfully built a comprehensive telemedicine system integrated across provincial, municipal, county, and township levels. At the same time, the price of telemedicine services has been reduced, and a price ceiling has been set up across the range of telemedicine services to the benefit of patients. Table 4 and Table 5 show how the government determined the price of telemedicine before and after the adjustment. MIs at all levels can set the price of telemedicine according to the guidance price.

By the end of 2018, telemedicine has expanded and covered the four levels of province, city, county, and township. Today, 291 public hospitals at or above the county level, and 1543 township hospitals and community health centers are connected to the telemedicine platform of Guizhou. There are 16,347 experts, 1352 remote diagnosticians, and 6239 imaging, ECG, and B-ultrasound technicians registered on the platform to provide telemedicine services. When Guizhou province began developing its telemedicine system in 2016, there were fewer than 100 instances in which telemedicine services were used. In 2018, this number had reached nearly 400,000. Among them were more than 40,000 disease teleconsultations, nearly 300,000 remote imaging diagnoses, more than 80,000 tele-cardiogram diagnoses, and 423 tele-training events reaching 458,000 trainees.

At present, a number of 321 public MIs in 14 poverty-stricken counties in Guizhou are incorporated into the province’s telemedicine system. In 2018, a total of 496,000 telemedicine services were provided across 14 counties, saving more than 7 million yuan in medical, transportation, accommodation, and other expenses. Guizhou has also explored and built a team of ‘long-stay’ experts. Each poor county has selected doctors above deputy senior level to form telemedicine expert service teams in county’s hospitals and conduct tele-consultations for township hospitals and village clinics. At present, a professional team of 496 consultants, 89 imaging diagnostic doctors, and 59 electrocardiographic diagnostic doctors are set up to cover 14 poverty-stricken counties so that people can access expert medical services at home.

To alleviate burdens on the poor, Guizhou has adjusted and improved the pricing of telemedicine services in public MIs, and stipulated that HMIs participating in counterpart assistance schemes should not charge tele-consultation fees for the hospitals they aid. At present, 25 provincial tertiary hospitals have exempted 66 poverty-stricken county hospitals from costs associated with telemedicine projects. At the same time, municipalities have reduced the cost of telemedicine services in hospitals in poverty-stricken areas. For example, in Qiandongnan prefecture, telemedicine charges were reduced by 10% on the basis of provincial pricing; in a deeply impoverished county such as Ceheng, county-wide public hospitals have carried out telemedicine projects at 85% of provincial pricing, meaning that the price of unidisciplinary and multidisciplinary tele-consultations were 85 yuan per visit to chief physicians and 68 yuan per visit to associate chief physicians.

The local government in Guizhou classifies and manages the prices of telemedicine services to promote their coverage across the province and share the high-quality medical resources between cities and rural MIs by remote manipulation. These efforts are designed to reduce the telemedicine operating costs of public MIs at all levels, ensure that the burden of disease (BOD) for residents is not increased, and provide satisfactory telemedicine for the people.

### 7.2. The Reimbursement Ratio of Telemedicine in Guizhou

After Guizhou province embedded telemedicine fees in medical insurance reimbursements, the reimbursement ratio of telemedicine expenses for patients in poverty-stricken counties ranged between 70% and 85%. On this basis, the reimbursement ratio of telemedicine for registered poverty-stricken households under the Filing Riser Policy increased by 5 percentage points, with 90% as the maximum reimbursement ratio. For example, in 2018, the total telemedicine fees for Qijiang County in Qiandongnan Prefecture was 728,400 yuan, while total medical insurance reimbursements amounted to 582,200 yuan. Self-payments amounted to 145,680 yuan and the reimbursement ratio was 80%. For patients in non-poverty-stricken counties, the reimbursement ratio of telemedicine expenses ranged from 55% to 65%. Based on the results of this study, we recommend that the reimbursement scope and ratio for residents in non-poverty-stricken areas should be increased to further promote telemedicine services in Guizhou.

### 7.3. The Initial Probability of (‘HMI Efforts’, ‘PMI Efforts’, ‘Patients’ Acceptance’) in Guizhou

The results of our social survey indicated that the initial intention of patients’ ‘acceptance’ strategy was higher than that of MIs (both PMIs and HMIs) ‘efforts’ strategy. The results showed that residents’ awareness of digital telemedicine policies was 72%, and that their willingness to accept telemedicine was 89%. In most cases, residents were aware of telemedicine policies through online media and government information. In addition, the majority (around 85%) of those who had already received telemedicine services were quite satisfied about the quality of the service provided. In relation to those who expressed their dissatisfaction about telemedicine services, the main reasons included high telemedicine fees; network security issues; and human–computer interactions leading to poor medical outcomes due to a lack of face-to-face communication. This is because, in the past, patients were used to traditional methods of diagnosis and preferred face-to-face consultations with a doctor. Nonetheless, the initial intention of residents in Guizhou, especially in remote areas, to accept telemedicine services was still high. However, the initial intention of MIs was not as high as that of patients and was lower than expected. In the survey, through interviews with doctors and administrators, it was discovered that in the practice of telemedicine, both the inviter and invitee have little motivation or incentive to change from traditional practices, given that they would not be paid differently regardless of their effort level, particularly MIs in poor areas such as Guizhou province. Therefore, in such places, the success of telemedicine is dependent more on the MIs than on the patients.

## 8. Discussion

By studying the initial intentions in the three scenarios in Section 5.1, it was found that the patients of all scenarios were the first to achieve ESS, with the evolution time being shorter than that of both PMIs and HMIs. Furthermore, for all the three groups, we conclude that the higher initial probability of strategy combination (‘HMI efforts’, ‘PMI efforts’, ‘patients’ acceptance’), the shorter evolution time. From a theoretical perspective, this conclusion is rational because patients have a higher acceptance probability due to the higher convenience, accessibility, and benefits they achieve from telemedicine compared to traditional alternatives. Indeed, the HMIs and PMIs can also save proportions of referral costs, e.g., costs related to the bi-directional referral between PMIs and HMIs. Although the patients have a higher acceptance willingness, the efforts put by HMIs and PMIS on the telemedicine is more important for the operation and quality of telemedicine. In the existing literatures, some researchers argued that the capacity of telemedicine service providers is more important for telemedicine success than the competence of the individuals receiving the service care [28,32,33]. Therefore, in the process of promoting telemedicine, the government should pay special attention to MIs and make full use of them. Considering financial incentives, to ensure that the benefits of MIs largely outweigh the costs, the government should adopt measures to improve the effort levels of MIs in the provision of telemedicine, which would thereby shorten their evolution time. Hofmann-Wellenhof et al. [34] examined the feasibility and acceptance of teledermatology for wound management of patients with chronic leg ulcers by home-care nurses, the results show that there was a significant decrease in visits to a general physician or the wound care center, and the acceptance of teledermatology was high in patients, home-care nurses, and wound experts. Telemedicine seems to be accepted both by patients and healthcare persons.

Through numerical simulation, it was concluded that if the cost of telemedicine is reduced, the evolutionary time of the tripartite game to reach ESS is shortened, which is beneficial for the promotion of the telemedicine system. In the same respect, it is unsurprising that patients will choose traditional face-to-face health service direct from HMIs via a long travel, if the telemedicine fees are comparatively much higher. Likewise, it is expected that if the telemedicine fees are so low, both HMIs and PMIs will not operate normally with less telemedicine income. Thus, it is obvious that the telemedicine has a rational range.

In fact, the changes in the reimbursement ratio of telemedicine fees are indirect changes in telemedicine fees. Therefore, if the reimbursement ratio increases, the telemedicine fees paid by patients decreases. Mapping together these findings, we can conclude that a higher reimbursement ratio will further promote the development of telemedicine. However, the numerical analysis suggests that the proportion of the medical insurance reimbursement ratio is neither as high as possible nor as low as possible; between 0.7 and 0.8 is the appropriate ratio for shorter evolution times. Although, it was found that telemedicine fees that are embedded in medical insurance reimbursements promote telemedicine more effectively than if they are not embedded. Thus, having the optimal reimbursement ratio is key for promoting telemedicine. Furthermore, if telemedicine service fees are not embedded within the scope of medical insurance reimbursement, the promotion of telemedicine is hindered because it increases the costs that patients have to pay.

This paper used evolutionary game theory to analyze behavioral strategies and their dynamic evolution in the implementation and operation of telemedicine and did numerical simulation by software Matlab with a view to developing management strategies that promote telemedicine as a new way of delivering health services. Although we found some implication of telemedicine promotion, there still work need to do in the future, e.g., the same as the important determinants of telemedicine cost, why reimbursement ratio of telemedicine fees have a turning point during value [0, 1], while among the appropriate domain, the lower the cost, the greater promotion of telemedicine, and the rational range of telemedicine fees also were not analyzed in this paper. Owing to the small sample, the reliability of questionnaire designed by our team are not bigger than 0.7. Furthermore, the survey was conducted in a small county of Qiandongnan Miao and Dong Autonomous Prefecture in Guizhou, therefore the case study in this paper may not be reflective of China or even other developing countries. In the future, we need to broaden our sample domain to do further research related to telemedicine system.

## 9. Conclusions

We found that the benefits being greater than the costs is the premise of implementing telemedicine. When this premise was satisfied, we further found that the higher initial probability of strategy combination (‘HMI efforts’, ‘PMI efforts’, ‘patients’ acceptance’), reducing telemedicine fees, and propriety reimbursement ratio range from 0.7 to 0.8 all contributed to better development of telemedicine.

Therefore, in the process of promoting telemedicine, the central Chinese government and local governments should pay attention to the operation of HMIs and PMIs and offer financial support when the costs are greater than the benefits. At the same time, the government should improve awareness of telemedicine and increase the participation of all three parties. Lastly, for effective telemedicine promotion, it is recommended that telemedicine services are incorporated within the scope of medical insurance and that the optimal reimbursement ratio is used.

## Figures and Tables

**Table 1:** The payoff matrix of higher medical institutions (HMIs), primary medical institutions (PMIs), and patients.

		HMIs	Efforts (x)		No Efforts (1−x)	
	PMIs					
Patients			Efforts (y)	No Efforts (1−y)	Efforts (y)	No Efforts (1−y)
Acceptance (z)			$E_{1}\left({\bar{e}}_{1}\right)+R_{1}\left({\bar{e}}_{1}\right)-C_{1}\left({\bar{e}}_{1}\right)$	$E_{1}\left({\bar{e}}_{1}\right)+R_{1}\left({\bar{e}}_{1}\right)-C_{1}\left({\bar{e}}_{1}\right)$	$E_{1}\left({\underline{e}}_{1}\right)+R_{1}\left({\underline{e}}_{1}\right)-C_{1}\left({\underline{e}}_{1}\right)$	$E_{1}\left({\underline{e}}_{1}\right)+R_{1}\left({\underline{e}}_{1}\right)-C_{1}\left({\underline{e}}_{1}\right)$
			$E_{2}\left({\bar{e}}_{2}\right)+R_{2}\left({\bar{e}}_{2}\right)-C_{2}\left({\bar{e}}_{2}\right)-\varepsilon B$	$E_{2}\left({\underline{e}}_{2}\right)+R_{2}\left({\underline{e}}_{2}\right)-C_{2}\left({\underline{e}}_{2}\right)-\varepsilon B$	$E_{2}\left({\bar{e}}_{2}\right)+R_{2}\left({\bar{e}}_{2}\right)-C_{2}\left({\bar{e}}_{2}\right)-\varepsilon B$	$E_{2}\left({\underline{e}}_{2}\right)+R_{2}\left({\underline{e}}_{2}\right)-C_{2}\left({\underline{e}}_{2}\right)-\varepsilon B$
			$E_{3}\left({\bar{e}}_{2}\right)-\left(1-b\right)B$	$E_{3}\left({\underline{e}}_{2}\right)-\left(1-b\right)B-G$	$E_{3}\left({\bar{e}}_{2}\right)-\left(1-b\right)B-G$	$E_{3}\left({\underline{e}}_{2}\right)-\left(1-b\right)B-G$
Non-acceptance (1−z)			$R_{1}\left({\bar{e}}_{1}\right)-C_{1}\left({\bar{e}}_{1}\right)$	$R_{1}\left({\bar{e}}_{1}\right)-C_{1}\left({\bar{e}}_{1}\right)$	$R_{1}\left({\underline{e}}_{1}\right)-C_{1}\left({\underline{e}}_{1}\right)$	$R_{1}\left({\underline{e}}_{1}\right)-C_{1}\left({\underline{e}}_{1}\right)$
			$R_{2}\left({\bar{e}}_{2}\right)-C_{2}\left({\bar{e}}_{2}\right)$	$R_{2}\left({\underline{e}}_{2}\right)-C_{2}\left({\underline{e}}_{2}\right)$	$R_{2}\left({\bar{e}}_{2}\right)-C_{2}\left({\bar{e}}_{2}\right)$	$R_{2}\left({\underline{e}}_{2}\right)-C_{2}\left({\underline{e}}_{2}\right)$
			0	0	0	0

**Table 2:** The eigenvalues of each equilibrium point’s (EP’s) *Jacobian matrix.*

EP	Eigenvalue		
$D_{1}\left(0,0,0\right)$	$R_{1}\left({\bar{e}}_{1}\right)-C_{1}\left({\bar{e}}_{1}\right)-R_{1}\left({\underline{e}}_{1}\right)+C_{1}\left({\underline{e}}_{1}\right)$	$R_{2}\left({\bar{e}}_{2}\right)-C_{2}\left({\bar{e}}_{2}\right)-R_{2}\left({\underline{e}}_{2}\right)+C_{2}\left({\underline{e}}_{2}\right)$	$E_{3}\left({\underline{e}}_{2}\right)-\left(1-b\right)B-G$
$D_{2}\left(0,0,1\right)$	$\begin{array}{c} E_{1}\left({\bar{e}}_{1}\right)-E_{1}\left({\underline{e}}_{1}\right)+R_{1}\left({\bar{e}}_{1}\right)\\ -C_{1}\left({\bar{e}}_{1}\right)-R_{1}\left({\underline{e}}_{1}\right)+C_{1}\left({\underline{e}}_{1}\right) \end{array}$	$\begin{array}{c} R_{2}\left({\bar{e}}_{2}\right)-C_{2}\left({\bar{e}}_{2}\right)-R_{2}\left({\underline{e}}_{2}\right)\\ +C_{2}\left({\underline{e}}_{2}\right)+E_{2}\left({\bar{e}}_{2}\right)-E_{2}\left({\underline{e}}_{2}\right) \end{array}$	$-\left(E_{3}\left({\underline{e}}_{2}\right)-\left(1-b\right)B-G\right)$
$D_{3}\left(0,1,0\right)$	$R_{1}\left({\bar{e}}_{1}\right)-C_{1}\left({\bar{e}}_{1}\right)-R_{1}\left({\underline{e}}_{1}\right)+C_{1}\left({\underline{e}}_{1}\right)$	$-\left(R_{2}\left({\bar{e}}_{2}\right)-C_{2}\left({\bar{e}}_{2}\right)-R_{2}\left({\underline{e}}_{2}\right)+C_{2}\left({\underline{e}}_{2}\right)\right)$	$E_{3}\left({\bar{e}}_{2}\right)-\left(1-b\right)B-G$
$D_{4}\left(0,1,1\right)$	$\begin{array}{c} E_{1}\left({\bar{e}}_{1}\right)-E_{1}\left({\underline{e}}_{1}\right)+R_{1}\left({\bar{e}}_{1}\right)\\ -C_{1}\left({\bar{e}}_{1}\right)-R_{1}\left({\underline{e}}_{1}\right)+C_{1}\left({\underline{e}}_{1}\right) \end{array}$	$-\left(\begin{array}{c} R_{2}\left({\bar{e}}_{2}\right)-C_{2}\left({\bar{e}}_{2}\right)-R_{2}\left({\underline{e}}_{2}\right)\\ +C_{2}\left({\underline{e}}_{2}\right)+E_{2}\left({\bar{e}}_{2}\right)-E_{2}\left({\underline{e}}_{2}\right) \end{array}\right)$	$-\left(E_{3}\left({\bar{e}}_{2}\right)-\left(1-b\right)B-G\right)$
$D_{5}\left(1,0,0\right)$	$-\left(R_{1}\left({\bar{e}}_{1}\right)-C_{1}\left({\bar{e}}_{1}\right)-R_{1}\left({\underline{e}}_{1}\right)+C_{1}\left({\underline{e}}_{1}\right)\right)$	$R_{2}\left({\bar{e}}_{2}\right)-C_{2}\left({\bar{e}}_{2}\right)-R_{2}\left({\underline{e}}_{2}\right)+C_{2}\left({\underline{e}}_{2}\right)$	$E_{3}\left({\underline{e}}_{2}\right)-\left(1-b\right)B-G$
$D_{6}\left(1,0,1\right)$	$-\left(\begin{array}{c} E_{1}\left({\bar{e}}_{1}\right)-E_{1}\left({\underline{e}}_{1}\right)+R_{1}\left({\bar{e}}_{1}\right)\\ -C_{1}\left({\bar{e}}_{1}\right)-R_{1}\left({\underline{e}}_{1}\right)+C_{1}\left({\underline{e}}_{1}\right) \end{array}\right)$	$\begin{array}{c} R_{2}\left({\bar{e}}_{2}\right)-C_{2}\left({\bar{e}}_{2}\right)-R_{2}\left({\underline{e}}_{2}\right)\\ +C_{2}\left({\underline{e}}_{2}\right)+E_{2}\left({\bar{e}}_{2}\right)-E_{2}\left({\underline{e}}_{2}\right) \end{array}$	$-\left(E_{3}\left({\underline{e}}_{2}\right)-\left(1-b\right)B-G\right)$
$D_{7}\left(1,1,0\right)$	$-\left(R_{1}\left({\bar{e}}_{1}\right)-C_{1}\left({\bar{e}}_{1}\right)-R_{1}\left({\underline{e}}_{1}\right)+C_{1}\left({\underline{e}}_{1}\right)\right)$	$-\left(R_{2}\left({\bar{e}}_{2}\right)-C_{2}\left({\bar{e}}_{2}\right)-R_{2}\left({\underline{e}}_{2}\right)+C_{2}\left({\underline{e}}_{2}\right)\right)$	$E_{3}\left({\bar{e}}_{2}\right)-\left(1-b\right)B$
$D_{8}\left(1,1,1\right)$	$-\left(\begin{array}{c} E_{1}\left({\bar{e}}_{1}\right)-E_{1}\left({\underline{e}}_{1}\right)+R_{1}\left({\bar{e}}_{1}\right)\\ -C_{1}\left({\bar{e}}_{1}\right)-R_{1}\left({\underline{e}}_{1}\right)+C_{1}\left({\underline{e}}_{1}\right) \end{array}\right)$	$-\left(\begin{array}{c} R_{2}\left({\bar{e}}_{2}\right)-C_{2}\left({\bar{e}}_{2}\right)-R_{2}\left({\underline{e}}_{2}\right)\\ +C_{2}\left({\underline{e}}_{2}\right)+E_{2}\left({\bar{e}}_{2}\right)-E_{2}\left({\underline{e}}_{2}\right) \end{array}\right)$	$-\left(E_{3}\left({\bar{e}}_{2}\right)-\left(1-b\right)B\right)$

**Table 3:** Local stability analysis results.

EP	Scenario 1 Costs Are More Than Revenue		Scenario 2 Costs Are Less Than Revenue	
	Eigenvalue Symbol	Stability	Eigenvalue Symbol	Stability
$D_{1}\left(0,0,0\right)$	−−−	Stable point	+++	Unstable point
$D_{2}\left(0,0,1\right)$	−−+	Saddle point	++−	Saddle point
$D_{3}\left(0,1,0\right)$	−+−	Saddle point	+−+	Saddle point
$D_{4}\left(0,1,1\right)$	−++	Saddle point	+−−	Saddle point
$D_{5}\left(1,0,0\right)$	+−−	Saddle point	−++	Saddle point
$D_{6}\left(1,0,1\right)$	+−+	Saddle point	−+−	Saddle point
$D_{7}\left(1,1,0\right)$	++−	Saddle point	−−+	Saddle point
$D_{8}\left(1,1,1\right)$	+++	Unstable point	−−−	Stable point

**Table 4:** The price of telemedicine services in Guizhou province.

Items	Unit	Price Ceiling (Yuan)		
		National Level	Provincial Level	Municipal/City Level
Tele-consultation	Hour	1550	700	595
Traditional Chinese Medical (TCM) tele-diagnosis and tele-consultation	Hour	1550	700	595
Synchronized tele-pathological consultation	Per visit	500	400	340
Asynchronized tele-pathological consultation	Per visit	400	300	255
Remote imaging conference	Per visit	400	200	170

Source: Guizhou Provincial Health Commission of Guizhou Province ‘Standardizing the price standard of telemedicine consultation service in our province’ (Implementation from 1 December 2015).

**Table 5:** The price of telemedicine services in Guizhou province after the price adjustment.

Items	Unit	Price Ceiling (Yuan)		
		National Level	Provincial Level	Municipal/City Level
Unidisciplinary tele-consultation	Per visit	Not exceed 100 per visit	100 for chief physician	100 for chief physician
		Not exceed 80 per visit	80 for associate chief physician	80 for associate chief physician
Multidisciplinary tele-consultation	Hour	1200	320	270
TCM tele-diagnosis and tele-consultation	Hour	1200	320	270
Synchronized tele-pathological consultation	Per visit	300	180	150
Asynchronized tele-pathological consultation	Per visit	300	140	120
Remote electrocardiogram (ECG) diagnosis	Per visit		The price is charged according to the current medical price of the inviting party of the ECG project in Guizhou Province	
Remote imaging diagnosis	Per visit			
Remote laboratory diagnosis	Per visit			
Telepathological diagnosis	Per visit			

Source: Guizhou Province Government ‘Guizhou Province adjusts and perfects the price scheme of telemedicine service projects in public MIs’ (Implementation from 1 July 2016).

## Abbreviations

ESS	evolutionarily stability strategies
EP	equilibrium point
MI	medical institution
HMI	higher medical institution
PMI	primary medical institution
TAM	technology acceptance model
TPB	theory of planned behaviour
